# The history of the Pan-African Society of Cardiology (PASCAR): the first 30 years, 1981–2011

**Published:** 2011-06

**Authors:** David A Watkins, Bongani M Mayosi, Samuel I Omokhodion

**Affiliations:** Department of Medicine, University of Cape Town, South Africa; Department of Medicine, University of Cape Town, South Africa; Division of Paediatric Cardiology, Department of Paediatrics, College of Medicine, University College Hospital, Ibadan, Nigeria

## Abstract

**Abstract:**

The year 2011 marks the 30th anniversary of the founding of the Pan-African Society of Cardiology (PASCAR). Throughout its brief history, PASCAR has been integral to improving the cardiovascular health of the people of Africa. During the past three decades, many African countries have been vulnerable to political and social turmoil, and PASCAR itself has been repeatedly challenged to press on with its mission, in spite of innumerable practical obstacles. This article celebrates the hard work and dedication of PASCAR’s founders and subsequent leaders, and challenges the present and future generations to carry on the charge of furthering the health of Africans.

## Foundation

The idea of PASCAR was conceived by a small group of African cardiologists during the late 1970s. At that time the World Congress of Cardiology was orientated towards cardiovascular conditions that were affecting the populations of Europe and North America. Many African cardiologists felt that they neither benefited nor were able to make much of an impact at these world meetings. In November 1979, Prof Ayodele Falase, then president of the Nigerian Cardiac Society, called upon the members of his Society to organise a continental meeting and inaugurate what he termed a ‘Pan-African Congress of Cardiology’. At the Nigerian Society’s meeting in Ibadan, Nigeria the following year, an organising committee was formed and funding was secured from pharmaceutical companies and the World Health Organisation, with administrative assistance from University College Hospital in Ibadan, Nigeria.

The first PASCAR congress was held in Badagry, Nigeria in May 1981. This meeting was most remarkable in the diversity of its participants. Over 120 clinicians and scientists from 15 African countries attended, and the event brought together English- and French-speaking Africans, fostering understanding between these historically separated groups. International collaboration and cooperation began in an unprecedented way, as cardiovascular workers were able to discuss their challenges, successes and research discoveries, and exchange ideas as never before. At the conclusion of the congress on 6 May 1981, the Pan African Society of Cardiology was officially inaugurated.

Out of this inaugural meeting, the organisation set for itself four goals: first, to prevent and treat cardiovascular disease in Africa; second, to educate and train African healthcare professionals about cardiovascular disease; third, to educate laypersons about heart disease; and fourth, to invest in cardiovascular research. The task ahead of PASCAR was monumental because in those days, it was generally accepted among local ministries of health that Africans had a ‘built-in protection’ against heart disease and that hypertension and other cardiac risk factors would never become epidemic on the continent.

## Early achievements

Over the next decade, PASCAR congresses began to gain international attention. In April 1983, the second congress was held in Nairobi, Kenya, with the aim to ‘take stock of different cardiovascular problems in the tropics’. The third congress, held jointly with the Egyptian Society of Cardiology in 1985, highlighted newer technologies such as echocardiography and cardiac conditions affecting younger people, such as rheumatic heart disease. During the same year, a very important meeting was convened in Harare, Zimbabwe, and attended by over 50 scientists and clinicians from across the world. This meeting established a collaborative research programme between American and African scientists and was funded by the US National Heart Lung and Blood Institute, the Association of Black Cardiologists (USA), and the International Society of Hypertension among Blacks. Out of this collaboration, many subsequent projects were seeded.

In March 1989, PASCAR convened for the fourth time and developed a governing structure for the organisation, including the creation of five regional bodies that would meet on a biennial basis. Importantly, this was the first meeting to recognise and declare hypertension as a major cause of morbidity and mortality among Africans. Following the success of that event, however, the next meeting (1991) had to be abandoned as it had been scheduled in Ethiopia, which at that time was in the throes of civil unrest.

In spite of the Ethiopian setback, the fifth PASCAR congress proceeded as planned in Yaoundé, Cameroon in April 1993. This particular meeting attracted an educational grant from the Rockefeller Foundation (USA) and various pharmaceutical companies and its theme was ‘preventative cardiology in Africa’. Over 500 specialists attended, including representatives of the American Heart Association, World Health Organisation, and International Society and Federation of Cardiology (later, World Heart Federation), making this the largest meeting to date.

## Stagnation

During the mid-1990s the pan-African meetings stagnated, in part due to political changes impacting on the organisation. For instance, in 1996 the General Secretary of PASCAR was named minister of health in Cote d’Ivoire. However advocacy efforts and regional meetings still continued. PASCAR members participated in the Organisation of African Unity conference of African ministers of health in 1995 and played a key role in highlighting the growing burden of chronic disease on the continent.

In February 1997, a workshop on hypertension and echocardiography was held in Yaoundé, supported by prominent American cardiologists such as Dr Richard Cooper, Dr Julian Haywood, and Dr George Mensah. In August of the following year, preparations began for the sixth congress, which commenced in August 1999 in Cotonou, Benin, under the leadership of Dr Hippolyte Agboton.

PASCAR entered a period of stagnation from 1998 to 2003; no PASCAR meetings were held during this period. Members of PASCAR were, however, becoming more prominently involved in international meetings, such as the American College of Cardiology, European Society of Cardiology, and the Asian Pacific Society of Cardiology. PASCAR had also been represented at the World Heart Federation meetings ever since that organisation’s inception.

## Revival

October 2004 marked the renaissance of PASCAR. The seventh congress successfully convened in Accra, Ghana and heralded the point at which the Society came out of hibernation, with discussions of constitutional reforms and a new executive committee. In keeping with this rebirth, the theme of the congress was ‘preventing cardiovascular disease in Africa: a time for concerted effort’. It was also an important meeting in terms of redefining the scientific mission of PASCAR: US cardiologist Dr Richard Cooper gave a well-received lecture on the cost effectiveness of research, and genetic research in particular. Similarly, the first talks on creating the *Cardiovascular Journal of Africa* were held.

The energy from the Accra congress was maintained into the following year. The first all-Africa workshop on rheumatic fever and rheumatic heart disease was held in 2005, leading to the so-called Drakensberg Declaration, which established Africaspecific policies for prevention and control of this disease.^1^,^2^ Further progress was made in establishing the academic activities and a journal for PASCAR. At the Drakensberg meeting, Prof Andries Brink initiated and led an effort to form the new *Cardiovascular Journal of Africa* as a journal for PASCAR, based on the established *Cardiovascular Journal of Southern Africa*, which he had founded 16 years before. This new journal quickly became the definitive academic cardiology journal on the continent and is accredited by all the world’s important databases. It is the most widely consulted source of information on cardiovascular disease in Africa.^3^

During the following years, PASCAR continued to grow in influence and reach on the African continent. In May 2007, the eighth PASCAR congress was held in Nairobi, Kenya. This meeting attracted over 300 participants from countries in all regions of Africa and beyond. A similarly global audience attended the ninth PASCAR congress in Abuja, Nigeria in September 2009. These conferences have confirmed the role of PASCAR as the premier umbrella association for national professional societies in cardiovascular medicine and surgery on the continent.

## A bright future

The fortunes of the Pan-African Society of Cardiology have been closely intertwined with the history of Africa herself. In this era of the African renaissance, inspired men and women have come forth, working together to address the cardiovascular health of their continent while celebrating their geographic, cultural and intellectual diversity. Despite the burdens of history and the immense task that lies ahead, PASCAR is full of vitality and is poised to lead the continent, and eventually the world, in finding solutions to the cardiovascular challenges of the 21st century.
